# Thriving from work questionnaire: German translation and validation

**DOI:** 10.1186/s12889-024-19037-0

**Published:** 2024-06-19

**Authors:** Stephanie M. Neidlinger, Susan E. Peters, Daniel A. Gundersen, Jörg Felfe

**Affiliations:** 1 Department of Work, Organizational, and Business Psychology, Helmut-Schmidt University, 22043 Hamburg, Germany; 2 Center for Work, Health, and Well-being, Harvard T.H. Chan School of Public Health, Boston, MA 02115 USA; 3 Rutgers Institute for Nicotine and Tobacco Studies, New Brunswick, NJ 08901 USA; 4 Division of General Internal Medicine, Rutgers Robert Wood Johnson Medical School, New Brunswick, NJ 08901 USA

**Keywords:** Questionnaires, Worker well-being, Item response theory, Study validation, Germany

## Abstract

**Background:**

The Thriving from Work questionnaire is a comprehensive indicator of positive well-being for employees, applicable in both research and practical contexts. Current discussions underline the crucial impact that employment should have in enriching workers’ lives positively and meaningfully, along with the necessity for accurate and dependable tools to assess employee well-being. This study investigated the reliability, validity, and dimensionality of the translated German adaptation of the Thriving from Work questionnaire developed by Peters and colleagues [1, 2]. The questionnaire assesses work-related well-being with 30 items clustered in six domains: emotional and psychological well-being, social well-being, work-life integration, physical and mental well-being, basic needs for thriving, and experiences of work.

**Methods:**

This study aimed to convert the Thriving at Work Questionnaire from English into German. We assessed the psychometric characteristics of the German version of the questionnaire by using item response theory with a sample of 567 German employees and examined its criterion validity.

**Results:**

We found that the long and short German Thriving from Work questionnaire versions are reliable with good construct validity. Criterion validity was demonstrated by relationships with important work and life outcomes, such as life satisfaction, trust in the organizations’ management, general well-being, work-related fatigue, and work stress.

**Conclusions:**

The current study demonstrated that the German language version of the questionnaire is both a reliable and valid measure of employee well-being. We discuss recommendations for further adaptation and future research.

**Supplementary Information:**

The online version contains supplementary material available at 10.1186/s12889-024-19037-0.

## Introduction

Thriving from Work (TfW) is a holistic and integrated concept of work-related well-being that captures a state of positive mental, physical, and social functioning in which employees’ experiences of their work and working conditions enable them to reach their full potential at work, home, and in the community [1, 2]. To assess the role of work for people’s overall happiness and well-being, instruments offering a broad conceptualization of work-related well-being are called for [3, 4]. TfW conceptualizes work-related well-being across six domains: (1) work-related emotional & psychological well-being, (2) social well-being from work, (3) work life integration, (4) basic needs for thriving, (5) job design and experience of work, and (6) health & physical and mental well-being from work. Various versions of the TfW questionnaire have been developed, tested, and validated in the U.S. and since then translated into Spanish and validated in Latin America [2, 5].

Existing instruments commonly used in European settings, such as COPSOQ [6] or the Burnout Assessment Tool BAT [7], often do not focus on resources but rather focus on work demands, strains, and undesirable consequences of work. Constructs measuring work-related well-being often consider resource expenditure rather than resources and their availability (e.g., Warwick-Edinburgh Mental Well-being Scale WEMWBS [8]) as contributing factors of work-related well-being or rely on comparative and/or affective statements describing well-being states (e.g., Subjective Happiness scale [9]). TfW adds to this conceptualization by addressing the role of work itself as a resource which can help organizations understand how work can be restructured to better enable overall positive employee well-being.

In the broader landscape of organizational psychology, TfW represents a new and pivotal concept encapsulating positive mental, physical, and social functioning within and from work and the professional environment and its cascading effects on life outside of work. By exploring Thriving from Work, this study contributes to a deeper understanding of work-related well-being, offering insights that can inform organizational practices and promote flourishing workplaces. Exploring the role of TfW in organizational psychology can offer a new perspective, potentially uncovering its significance as both a predictor and an outcome in workplace research.

The purpose of this study is to translate the TfW questionnaire (TFWQ) into German and conduct a comprehensive validation using item response theory to ensure its reliability and effectiveness in assessing work-related well-being in the German context. Our study adds to the literature on work-related well-being in two ways: First, we provide a translation and validation of the English language TfW questionnaire. The questionnaire is currently available in English and Spanish; a German validation thus allows for the application of the questionnaire with German speaking employees. Second, we used item response theory to conduct the validation, thus employing methodological rigour to our study.

## Theoretical background

### Theoretical foundations of the instrument

TfW is a model for work-related well-being, featuring six distinct dimensions [1]:

1. Psychological and emotional well-being from work refers to the sense of meaning and purpose in one’s job, the opportunities for personal growth and development, and the alignment of individual values with the company’s mission and values.
2. Social well-being from work involves supportive relationships, a sense of being valued and belonging, respectful treatment, contributions to others, fair treatment, and recognition within the workplace.
3. Work-life integration relates to achieving a balance between work and personal life, including considerations of commuting and work-family responsibilities.
4. Basic needs for thriving from work encompass job security, fair pay, employee benefits, and opportunities for career advancement.
5. Job design and experience of work involve elements such as autonomy, access to adequate resources, skills and knowledge, and managing work intensity.
6. Health, physical, and mental well-being from work includes factors like physical and psychological safety at work, and prevention of exhaustion.

### Development of the instrument

The TfWQ was developed using an iterative participatory approach with three main research activities. First, in 2019, the lead investigators conducted a 90-minute workshop with 33 multi-disciplinary experts whom had conducted research and/or practice in worker well-being to obtain a definition and conceptualization of TfW. Then the investigators mapped potential items drawing from reliable and valid instruments as well as investigator designed items, when there were no reliable of valid instruments to draw from, which were subsequently reviewed and refined [1]. Second, the research team conducted interviews with 18 expert researchers to obtain feedback on the first draft of the questionnaire in an iterative fashion, making changes to the items before obtaining further expert input. Third, the investigator team conducted four rounds of cognitive testing [10] with a diverse range of employees with different occupational and demographic characteristics and literacy levels using the think-aloud method with retrospective probing.

Since the TfW questionnaire consists of six dimensions and two stand-alone items, it can thus be considered a multifaceted construct. Moreover, Peters and colleagues [2] conceptualized TfW as a bifactor model. The bifactor model approach [11] simultaneously assesses the construct’s general effect/ factor of TfW as well as specific effects/ factors of the six domains. This model assesses each item’s relationship with both the general TfW construct and its specific domain, ensuring all items load onto both the general and specific domains as hypothesized. For more information on the development of the items and dimension structure, refer to Peters and colleagues [2]. They stated that the six domains were conceptually relevant but not initially intended to be used on their own to measure these domains independently. This is for two main reasons: (a) to improve the reliability of each of the dimensions more items would need to be added which would include items that do not load as strongly onto the latent TfW construct causing a further limitation of increasing questionnaire length without improving the measurement of TfW, (b) the dimensions of TfW have further utility in identify priority areas for improving employee well-being. Further research is being conducted by Peters and colleagues to better measure the domains of TfW using stand-alone instruments to enable researchers and practitioners to have a reliable and valid instrument if the measurement of a TfW domain such as social well-being from work is desired. For more information on the conceptualization of the dimension of TfW and the rationale for the bi-factor model structure refer to Peters and colleagues [1, 2].

### Similarities and differences compared to existing instruments

In terms of assessed constructs, the concept of TFW displays some similarity with current measures of work-related happiness (e.g., SHS [9]), overall job satisfaction (e.g., COPSOQ [6]), and work engagement (e.g., UWES [7]). There is also a scale with a similar name measuring Thriving *at* Work developed by Porath and colleagues [12], which has a narrower focus that measures the effects of work on learning and vitality. The TfWQ differs to these instruments as it (1) addresses multiple dimensions of employee well-being, (2) measuring domains not previously considered in scales measuring work-related well-being (e.g., “job design and experience of work”), and (3) focuses on how work contributes to well-being in a positive way. The TfWQ also differs from the many instruments used as proxies for employee well-being, such as burnout and strain (Burnout Assessment Tool BAT [13]). Furthermore, resource expenditure is more often considered over its positive contribution to well-being (e.g., Warwick-Edinburgh Mental Well-being Scale WEMWBS [8]).

Established findings on work-related well-being often draw on the job-demands resources (JD-R) model [14]. In the JD-R model, job resources are crucial for buffering the impact of job demands on employee strain and fostering motivation and engagement, whereas job demands lead to negative outcomes. TfW could be conceptualized as an overarching outcome of the JD-R model, reflecting the positive effects of job resources on employee well-being whilst simultaneously taking some job demands into account. The introduction of the concept of TfW can make a valuable contribution to both the JD-R model and the broader field of organizational psychology in two ways: Firstly, TfW offers a holistic perspective on well-being at work, capturing not only psychological aspects but also physical and social dimensions, thus recognizing the multidimensional nature of well-being. Secondly, TfW can expand the scope of the JD-R model by emphasizing positive outcomes beyond mere job satisfaction or engagement.

### Validation of the U.S. English TfW questionnaire

The English language TfW questionnaire was validated with two samples of 1550 (calibration sample) and 500 (validation sample) employees across different sectors in the United States. Empirical reliability for both the long and the short form was high (0.93 and 0.87 respectively). Retest reliability was examined in a random sample of 100 employees from the validation sample via intra-cluster correlation coefficients for the long and short form model a-posteriori for participants in the validation and retest sample. Peters and colleagues [2] reported an intra-cluster correlation of 0.89 and 0.84 for the long- and short-forms, thus establishing good retest reliability.

Construct validity was assessed by examining the relationships between TfW and established constructs (e.g., Cantril’s Thriving Ladder, general life satisfaction, mental health [2]). In a subsequent Spanish validation study of the TfW questionnaire [5], community well-being, flourishing, vitality, meaning and purpose and organizational leadership were examined to ensure construct validity. TfW had moderate to high correlations with all validation constructs as hypothesized by the authors.

### Aims of the current study

The present study establishes a German translation of the TfWQ. Previous instruments available in the German language are not as comprehensive, often overlooking crucial domains such as job design and the experiential aspect of work, and failing to highlight the positive contributions of work to well-being. The goal with the German TfW questionnaire is to provide German speaking researchers and practitioners with an empirically validated measure of work-related well-being. The aims of this study were to evaluate the dimensionality, reliability, and validity of a translated German version of the TfWQ, and provide recommendation for its use in German speaking employee populations.

## Methods

### Sample

567 employees from diverse occupational and demographic backgrounds in Germany completed a one-time survey between February and June 2023. The sample was recruited via a market research institute which provided a small financial incentive for participation, as well as via personal connections of the researchers and their student assistants (no financial incentive). Participants reported a mean age of *M* = 31.46 years (*SD* = 9.17). 44.10% of the sample identified as male. Participants were balanced in terms of white collar and brown collar employees. Most participants worked in the armed forces and police (29.8%), IT or telecommunications (7.4%), nursing, medicine, and health (7.2%) and public administration (6.9%). The sample was highly educated, 31.75% of participants completed high school, 31.04% held a bachelor’s degree, 21.69% held a master’s degree, 14.64% completed vocational training, and 0.88% had completed their PhD/MDs. 25.05% of participants reported having managerial/ leadership roles.

The market research institute which assisted us in recruiting participants unfortunately did not provide the researchers with non-response rates. Since participants received a small financial incentive for completion of the questionnaire only upon full completion, we expect the non-response rate to be very low. Out of participants which were recruited from the researchers and their students’ personal networks, 95.25% of participants who opened the questionnaire completed it in full. The 4.75% of remaining participants opened the questionnaire but never started it.

### Translation of the questionnaire

To translate the TfWQ from English to German, we performed a forwards and backwards-translation approach with two bilingual translators as described by Beaton and colleagues [15]. The forward- and back-translated versions of the questionnaire were reviewed, and discrepancies were discussed and resolved by the research team, two of which were bilingual.

### Variables and measures

*Thriving from Work.* TfW was measured using the translated TfWQ. The German translation can be found in Appendix A. Respondents were asked to rate each item on a 6-point Likert scale, ranging from “never” (0) to “always” (5). Some example items of the 6 domains include statements such as “My job allows me to reach my full potential” (Psychological and Emotional Well-being from Work), “I am treated with respect in my workplace” (Social Well-being from Work), “I can achieve a healthy balance between my work and my life outside of work” (Work/life Integration), “I have good opportunities for promotion” (Basic Needs for Thriving), “I have access to the resources I need to do my job well” (Experience of Work and Job Design), and “I feel psychologically safe at work” (Health and Physical and Mental Well-being from Work). There are two additional items that load only onto the general factor of Thriving form Work, “I can voice concerns or make suggestions at work without getting in trouble” and “I receive recognition at work for my accomplishments”. The instrument can be used in the long or short form with 30 and 8 items, respectively. For the short form, Peters and colleagues [2] selected the items which showed the highest marginal discrimination parameter on the general TfW factor. Empirical reliability was 0.93 for the long form was and 0.86 for the short form.

### Validation measures

Life satisfaction, trust in management, well-being, fatigue, and stress were examined to assess convergent validity. An item that asked participants about phone use at work assessed discriminant validity.

*Life Satisfaction.* Satisfaction with life was assessed with five items from the SWLS (Satisfaction with life scale [16]). All items were ranked on a 7-point scale ranging from (1) strongly disagree to (5) strongly agree. A sample item was” The conditions of my life are excellent.” Cronbach’s alpha was 0.90.

*Well-being.* Well-being was assessed with the WHO-5 five-item well-being index [17]. All items were ranked on a 5-point scale ranging from (1) do not agree at all to (5) completely agree. An exemplary statement was “My daily life has been filled with things that interest me”. Cronbach’s alpha was 0.91.

*Trust in Management.* Participant’s trust in their organizations’ management was assessed with a single item from NIOSH Well-BQ [18] ranked on a 7-point scale ranging from (0) never to (6) always. The item was “I trust the management at my organization”.

*Fatigue.* Participant’s fatigue was assessed with a single item from NIOSH Well-BQ [18] ranked on a 7-point scale ranging from (0) never to (6) always. The item was “How often do you experience fatigue when you are working?”.

*Stress at work.* Participant’s stress was assessed with one item from NIOSH Well-BQ [18] ranked on a 7-point scale ranging from (0) never to (6) always. The item was “How often did you experience stress with regard to your work?”.

*Phone calls.* Participant’s phone calls were assessed using a single investigator-developed item: “In the last 4 weeks, to what extent have you used a telephone for work-related purposes?”. Responses were provided on a 5-point scale ranging from (1) never to (5) always.

### Methodological approach

The original English language version of the TfWQ was conceptualized as a bifactor model [1, 2]. As such, we validate the German language version fitting a bifactor model using the item response theory (IRT) framework. A bifactor IRT model has been established as an equivalent statistical model to confirmatory factor analysis (CFA) [19] but parametrized with unstandardized factor loadings – referred to in the IRT field as discrimination parameters [20]. There are benefits to using IRT as it allows for a closer examination of the relationship between someone’s trait level and their expected responses to each item, as well as the item-level contribution to conditional measurement precision. Because measurement precision is conditional on trait level in IRT, we present the empirical reliability as an estimate of the marginal reliability [21]. The empirical reliability is a measure of the ratio of true score variance to the true + error variances. Since the IRT bifactor model is equivalent to a bifactor CFA model [19] we used the model fit indices popular in the classical test theory and structural equation model literature.

The general factor of TfW represents the common variance shared among all items in the questionnaire. It captures the overall construct or latent trait of *TFW* and accounts for the facets’ shared commonality. In addition to the general factor, specific factors are introduced to account for the unique variance associated with subsets of items forming a facet of the latent construct with a unique influence on and beyond the general factor [22]. The specific factors are not intended to be used separately at this point (although, further research is being conducted on this by the authors); the statistical underpinning of our model may thus appear unifactorial due to its conceptualization. Bifactor models warrant a different analytic approach to assess their validity than the standard approach of CFA due to higher complexity, item-level analysis, and local dependence of all items [11, 22]. The bifactor model allows us to discern the nature of residuals associated with each specific factor, shedding light on the unique variance of the specific factors while simultaneously accounting for shared variance [11]. When our bifactor model is specified correctly, it achieves a more accurate representation of the latent structure of *TfW* as suggested by [2].

### Statistical analysis

We evaluated the psychometric properties of the German TfW questionnaire using the same approach in the original development and validation studies [2], and in a Spanish validation conducted in Peru and Mexico [5]. First, we examined the responses at the item level to identify any items that lacked variability, were more/ less difficult or showed low standard deviations. Second, we examined the intercorrelations between all items. We examined the empirical reliability as an indicator of scale reliability for both the general TfW construct and for each specific domain. Thirdly, we used IRT [23] models to analyze the long- and short- versions of the questionnaire. Logistic approximation was used to calculate the marginal discrimination parameters for the long-form. For the short-form, a unidimensional graded response IRT model was employed with all items loading on to the general (G) TfW factor.

We assessed the fit of the statistical model employing common criteria such as Root mean square error of approximation (RMSEA), Standardized Root Mean Squared Residual (SRMSR), and Comparative Fit Index (CFI), which are established metrics for model evaluation rooted in classical test theory and structural equation modelling. However, it is important to emphasize that cut-off values for these metrics are considered arbitrary in the field and do not represent rigid thresholds for model assessment [24, 25] and should be interpreted considering the theoretical and practical implications for the instrument. In recent years, cut-off values have been transferred to IRT models without adaptation. Consequently, relying solely on these traditional cutoffs can result in unreliable assessments of IRT model fit. To make our approach more comprehensive, we took into account all available metrics to make a balanced judgment about the suitability of the models [25]. In our case, this consisted of additional parametric bootstrapping simulations to determine if the model fits the data well, if cut-off values for traditional model fit indices are not met.

Lastly, since the selected statistical approach of a bifactor model with a general factor and the six specific factors was driven by theory, we refrained from testing the resulting model against competing models. All analyses were implemented using the mirt package in R [26]. A meta-analysis by Morin and colleagues [27] indicates that the bifactor model typically shows better fit than other statistical models like CFA, exploratory structural equation modeling (ESEM), and their hierarchical variants. This superior fit statistically supports the use of the bifactor model when it aligns with the theoretical framework of a study as was outlined in the original conceptualization paper by Peters and colleagues [1]. The selection of a bifactor model was therefore judged based on its theoretical coherence and empirical performance.

## Results

Appendix A contains the final long and short-form German TfW questionnaire.

Table 1 shows the distributions for each item of the long-form. Items tended to skew towards the higher responses (i.e., usually to always). Item 26 and 28 demonstrated reduced response variability. Intercorrelations of these two items with the rest of the questionnaire were low. Item 26 had an average correlation of 0.28 with the rest of the questionnaire and item 28 showed an average correlation of 0.11; thus, we suggest that these two items may be omitted from the German version of the questionnaire without disruption to the model fit. The full tables displaying descriptive statistics, as well as all polychoric item intercorrelations are displayed in Appendix B.

**Table 1:** Item distributions

Item		never	rarely	sometimes	usually	almost always	always
**Work-related Emotional & Psychological Well-being**							
1	I love my job.	6.00	9.35	20.28	24.16	28.57	11.64
2	My work adds meaning to my life.	5.82	15.17	17.81	26.10	23.81	11.29
3	My job allows me to achieve my full potential.	6.17	16.23	23.81	25.22	21.69	6.88
4	The kind of work I do makes me happy.	3.53	13.40	21.87	23.99	27.34	9.88
5	I am satisfied with my job.	2.12	9.70	15.87	28.40	33.33	10.58
6	My work adds to my overall life satisfaction.	4.06	13.23	20.63	27.16	26.10	8.82
**Social Well-being from Work**							
7	I am treated fairly at work.	1.06	5.47	11.82	27.16	33.86	20.63
8	I feel supported by the people I work with.	1.23	6.70	17.11	26.81	33.16	14.99
9	I feel valued by the people I work with.	1.76	7.41	14.99	26.28	31.57	17.99
10	I am treated with respect at work.	1.06	3.53	9.35	21.34	36.68	28.04
11	At work, I feel like I belong.	1.76	7.58	13.23	24.34	33.16	19.93
**Work-life Integration**							
12	I can achieve a healthy balance between my work and my life outside of work.	3.88	11.99	19.93	23.63	26.46	14.11
13	I can easily manage my job as well as attend to my needs and the needs of my family.	2.65	12.87	23.63	27.87	22.75	10.23
14	I feel safe getting to and from work.	0.35	3.35	4.94	14.64	30.16	46.56
**Basic Needs for Thriving**							
15	I am paid fairly for the job I do.	7.58	7.05	12.35	20.28	25.57	27.16
16	I am satisfied with the amount of paid leave I can take to care for myself or family members.	6.00	9.35	14.29	21.16	26.98	22.22
17	I feel my job is secure.	1.76	3.53	7.05	14.81	30.16	42.68
18	I have good opportunities for promotion.	8.47	17.28	13.40	28.75	19.93	12.17
**Job Design & Experience of Work**							
19	I am happy with how much input I have in decisions that affect my work.	4.23	14.64	17.64	28.40	27.51	7.58
20	I can easily manage the demands of my job.	0.71	3.17	19.22	33.86	31.39	11.64
21	I have adequate control over the pace of my work.	1.94	7.76	15.52	28.40	33.33	13.05
22	I am happy with how much control I have over my work schedule.	1.76	7.58	14.46	26.10	33.86	16.23
23	I have access to the resources I need to do my job well.	1.06	3.70	12.70	27.34	36.68	18.52
**Health, Physical, and Mental Well-being from Work**							
24	I feel psychologically safe at work.	2.65	9.70	14.11	23.28	29.45	20.81
25	I feel physically safe at work.	1.59	2.29	6.17	17.11	25.40	47.44
26	I feel excessive levels of stress from my work.**	3.70	14.29	16.58	33.16	27.16	5.11
27	After I leave work, I have enough energy to do the things I want or need to do.	3.88	13.93	22.57	30.69	21.34	7.58
28	I worry that I will get hurt at work.**	0.88	4.41	7.76	8.29	25.93	52.73
29	I can voice concerns or make suggestions at work without getting into trouble.	1.94	6.17	16.05	23.81	29.81	22.22
30	I receive recognition at work for my accomplishments.	3.88	14.46	21.34	25.93	23.99	10.41

Note: *N* = 567. All values indicate %. Items marked ** need to be reverse-coded for analysis. Items 29 and 30 only load on the general factor

Table 2 shows marginal discrimination parameters for the general factor of TfW and for each of the six domains. To test whether items performed consistently across individuals, marginal discrimination parameters were examined. Items with a high discrimination parameter are sensitive to changes in the latent construct whereas items with low discrimination parameters are less sensitive to latent changes. Discrimination parameters were mostly in the moderate to low range based on the typical thresholds of low (< 0.3) and moderate (around 0.3 to 1.0). The marginal discrimination parameters for Psychological and Emotional Well-being ranged from 0.78 to 1.24; Social Well-being from Work from 0.47 to 1.12; Work-life Integration: − 0.27 to 1.17; Basic Needs: 0.45 to 1.72; Experience of Work & Job Design from − 0.13 to 1.24; and, for Physical and Mental Well-being and Safety from − 0.24 to 1.16.

**Table 2:** Marginal discrimination parameters

#	Item	General Thriving from Work	1	2	3	4	5	6
**Work-related Emotional & Psychological Well-being**								
1	I love my job.	1.5069	1.1484					
2	My work adds meaning to my life.	1.2746	1.2375					
3	My job allows me to achieve my full potential.	1.4807	1.0492					
4	The kind of work I do makes me happy.	1.7427	1.1635					
5	I am satisfied with my job.	2.1049	0.7825					
6	My work adds to my overall life satisfaction.	1.7914	0.9867					
**Social Well-being from Work**								
7	I am treated fairly at work.	2.0666		0.4727				
8	I feel supported by the people I work with.	1.8796		0.7104				
9	I feel valued by the people I work with.	2.0105		1.1238				
10	I am treated with respect at work.	2.2489		0.6081				
11	At work, I feel like I belong.	1.7561		0.5384				
**Work-life Integration**								
12	I can achieve a healthy balance between my work and my life outside of work.	1.6960			1.1741			
13	I can easily manage my job as well as attend to my needs and the needs of my family.	1.7671			0.9399			
14	I feel safe getting to and from work.	1.1532			–0.2705			
**Basic Needs for Thriving**								
15	I am paid fairly for the job I do.	0.9170				1.7205		
16	I am satisfied with the amount of paid leave I can take to care for myself or family members.	1.2885				0.6002		
17	I feel my job is secure.	0.9546				0.4530		
18	I have good opportunities for promotion.	0.9246				0.5601		
**Job Design & Experience of Work**								
19	I am happy with how much input I have in decisions that affect my work.	1.7822					-0.1323	
20	I can easily manage the demands of my job.	1.2806					0.9470	
21	I have adequate control over the pace of my work.	1.4136					1.2423	
22	I am happy with how much control I have over my work schedule.	1.7971					0.5379	
23	I have access to the resources I need to do my job well.	1.5089					0.2008	
**Health, Physical, and Mental Well-being from Work**								
24	I feel psychologically safe at work.	1.9525						0.2456
25	I feel physically safe at work.	1.2012						1.1591
27	After I leave work, I have enough energy to do the things I want or need to do.	1.5470						-0.2387
29	I can voice concerns or make suggestions at work without getting into trouble.	1.7348						
30	I receive recognition at work for my accomplishments.	2.0583						
	*Empirical reliability*	0.9321	0.7574	0.6078	0.6234	0.6222	0.5972	0.3925

Note: *N* = 567. M2 _(df = 211)_ = 653.29; *p* < .001; RMSEA = 0.06; SRMSR = 0.06; CFI = 0.91. The last two items are not associated with a domain and only load to the general factor. Items 26 and 28 were dropped for this analysis. 1 = Psychological Emotional Well-being from Work, 2 = Social Well-being from Work, 3 = Work-Life Integration, 4 = Basic needs for Thriving, 5 = Experience of Work & Job Design, 6 = Physical & Mental Well-being and Safety. MHRM estimator was used

Classical test theory would expect all items of the specific factors (domains) to display higher loadings onto their respective domains. Peters and colleagues [2] conceptualized all items of TfW to load first onto the general TfW factor to identify the most important items for supporting employees’ thriving. They stated that the six domains were conceptually relevant but not initially intended to be used on their own to measure these domains independently.

The marginal discrimination parameters can display negative or lower values for the domains, as long as they show adequate loading onto the general factor. Additionally, when assessing the empirical reliability of TfW, the general factor’s empirical reliability should be assessed, rather than the specific factors’ reliabilities. The general factor of TfW displayed a high empirical reliability of 0.93 confirming its ability to reliably measure the shared variance among all items. Five of the specific factors (S1, S2, S3, S4, S5) exhibited moderate reliabilities, ranging from 0.60 to 0.76, affirming their capacity to reliably capture the unique variances associated with each dimension. However, one specific factor, S6, displayed a comparatively lower reliability of 0.40. While this may warrant closer examination in the future, the high general factor reliability underscores the instrument’s overall robustness in effectively measuring the latent construct of TfW.

Both marginal discrimination parameters and empirical reliabilities of the general and all of the specific factors were comparable to the original U.S. English language validation study [2] and the Spanish language validation study [5]. Therefore, a consistent picture emerges for the conceptualization of TfW across English, Spanish and German language samples.

RMSEA and SRMSR both yielded values of 0.06, suggesting a good fit of the model, while the CFI of 0.91 indicated substantial improvement over a null-model. While the chi-square test suggested some lack of fit as is often found in practical applications, the RMSEA, SRMSR, and CFI collectively suggest that the model provided a reasonably good representation of the data. Parametric simulations reproduced the data well, further supporting good model fit to the data.

### Validation of the short form

Table 3 shows the discrimination parameters and intercepts of the eight short-form items. Discrimination parameters ranged from 0.94 to 2.26. Item 15 (“I am paid fairly for the job I do”) displayed the lowest discrimination parameter of 0.94. This, however, is still above the usual cutoffs used in classical test theory and we concluded that no discrimination parameter posed a threat to the short-form’s model quality.

**Table 3:** Short-form discrimination parameters and intercepts

Long form item #		Discrimination Parameter	Category intercepts				
			1	2	3	4	5
1	I love my job.	1.59	3.72	2.40	0.84	-0.57	-2.77
7	I am treated fairly at work.	2.26	6.80	4.24	2.51	0.30	-2.31
12	I can achieve a healthy balance between my work and life outside of work.	1.52	4.15	2.28	0.87	-0.47	-2.43
15	I am paid fairly for the job I do.	0.94	2.82	2.00	1.11	0.08	-1.18
19	I am happy with how much input I have in decisions that affect my work.	1.79	4.35	2.22	0.91	-0.87	-3.57
20	I can easily manage the demands of my job.	1.19	5.60	3.72	1.44	-0.39	-2.50
24	I feel psychologically safe at work.	1.93	5.12	3.01	1.66	0.04	-2.06
29	I can voice my concerns or make suggests at work without getting into trouble.	1.69	5.17	3.42	1.73	0.15	-1.82

Note: Empirical reliability = 0.86. Model fit: C2_(df=20)_ = 729.9; RMSEA = 0.09; SRMSR = 0.06; CFI = 0.96.

The empirical reliability was 0.86, indicative of reasonably consistent and reliable measurements. RMSEA was 0.09, exceeding conventional cutoff values. The SRMSR was 0.06 indicating reasonably good fit, while the CFI was high (0.96) demonstrating a strong degree of fit. To further assess model fit, we conducted parametric bootstrapping simulations for each of the short-form items. All observed values were within the 2.5% and 97.5% confidence bounds indicating good model fit (Appendix C).

Peters and colleagues [2] stated that the short form of the questionnaire can be used with either item 24 (psychological safety) or 25 (physical safety), depending on the sample’s occupational characteristics and focus of interest. We thus ran all short-form analyses using item 24 (psychological safety) and item 25 (physical safety) interchangeably. Discrimination parameters and model fit indices were comparable for both models regardless of which item is used (short-form with item 25: Empirical reliability = 0.85. Model fit: C2 (*df* = 20) = 122.6; RMSEA = 0.10; SRMSR = 0.06; CFI = 0.95).

To enhance the accessibility of our findings for readers who are more accustomed to CFA, we have reported the loadings of our IRT discrimination parameters to the general and specific factors which can be interpreted similarly to CFA factor loadings for both the long and short forms of the questionnaire in Appendix D.

### Convergent and divergent validity

We tested the correlations between model-based scores of TfW with the validation constructs. All correlations were of similar strength and in the expected direction. Table 4 shows the correlations between the TFW short- and long- version with the validation constructs.

**Table 4:** Correlation between model-based scores for TfW with the validation constructs

		M	SD	1	2	3	4	5	6	7	8	9	10
1	Age	31.46	9.17	–									
2	Gender	44.10% male		− 0.03	–								
3	Education	–		0.13**	0.02	–							
4	*TfW* long from	–		0.03	− 0.07^†^	0.02	–						
5	*TfW* short from	–		0.02	− 0.09*	0.03	0.96***	–					
6	Life Satisfaction	4.75	1.27	− 0.08^†^	− 0.07	0.03	0.65***	0.64***	–				
7	Trust in Management	2.09	0.83	0.04	0.02	0.03	0.59***	0.60***	0.33***	–			
8	Well-being	3.54	1.10	0.09*	− 0.14***	0.08^†^	. 64***	0.60***	0.66***	0.36***	–		
9	Fatigue	4.44	1.34	− 0.19***	0.11**	− 0.03	− 0.41***	− 0.39***	− 0.33***	− 0.28***	− 0.53***	–	
10	Stress	3.41	1.16	− 0.08*	0.11**	0.04	− 0.49***	− 0.48***	− 0.34***	− 0.32***	− 0.46***	0.44***	–
11	Phone calls	3.25	1.30	0.12**	− 0.04	0.04	0.08^†^	0.07^†^	0.07	0.02	0.09**	0.01	− 0.01

Note. Gender was coded 1 = male, 2 = female, 3 = other (option 3 was selected by only 1 participant). TfW was ranked on a 6-point scale ranging from 1–6. Life Satisfaction, Trust in Management and Stress were ranked on a 7-point scale ranging from 1–7. Trust in Management was ranked on a 7-point scale ranging from 0–6. Well-being and Phone calls were ranked on a 5-point scale ranging from 1–5

TfW was positively associated with life satisfaction, trust in management and well-being and negatively associated with fatigue and stress. As expected, there was no significant relationship between TfW and using a phone for work. All relationships were in the expected direction and of the expected magnitude supporting that the questionnaire has acceptable discriminant and convergent validity. The long and short form of the questionnaire were correlated at 0.96***.

Peters and colleagues [2] recommend researchers use model-based soring for both long and short form of the TfW questionnaire. However, to make the questionnaire accessible for practitioners without advanced statistical knowledge, they recommend a standardized sum scoring approach. In Appendix E, we reported the correlations between the validation constructs and the sum scores of TfW.

## Discussion

The TfW questionnaire measures work-related well-being [2]. Within this study, we showed that the German translation and adaptation of the TfW questionnaire is a reliable and valid measure of work-related well-being that can be applied in the German context.

Two items assessing excessive stress from work (item 26) and the risk of injury (item 28) did not perform as expected (compared to the English language original validation). This might be due to the strict labor laws in Germany which increased safety at work to very high levels or the item might have been interpreted differently than in the U.S./ English validation study. Item 28 also did not perform as expected in the recently completed Spanish validation study [5], thus showing some consistency in our findings. Therefore, we suggest that these items could be removed from the German version of the questionnaire, making the German TfWQ an instrument with 28 items.

We found the German short and long-versions of the TfWQ were valid and reliable measure for work-related well-being and demonstrated good model fit. Fit indices were within the usual ranges for reliable instruments and additional parametric bootstrapping simulations provided additional support for model fit (Appendix C).

Findings for reliability and validity were similar to both the original validation study (Peters et al., 2023) and the Spanish translation and validation [5]. We expected TfW to show strong positive relationships with life satisfaction, trust in management, and well-being. Due to the conceptualization of TfW as a holistic construct measuring work-related well-being which extends to life outside of work, the high correlations between TfW and life satisfaction and well-being were to be expected as there is conceptual overlap. We expected trust in management to be an important antecedent of work-related well-being, as previous research has shown associations between these two constructs [28]. When employees have low trust in their organization’s management, thriving from their work may be reduced. We recommend future researchers to examine this relationship in longitudinal studies to examine causality. We further expected TfW to have strong negative relationships with fatigue and stress as previous studies reported negative associations between these constructs with other measures of well-being at work [29, 30]. We included the frequency of participants’ work-related phone calls to assess discriminant validity. As most jobs require communication by phone, we did not expect there to be any relationship between TfW and phone calls. This assumption was supported by our data.

### Recommendations for the use of the German thriving from work questionnaire for both researchers and practitioners

It is important to consider context and culture-specific factors when adapting questionnaires. Within our analyses, two items (item 26: “I feel excessive levels of stress from my work.” and item 28: “I worry that I will get hurt at work”) were found to have reduced response variability and low intercorrelations with the rest of the items. After careful consideration of the German cultural context with its comparatively strict laws regarding employee health and safety, the two items were excluded from further analysis as they were found to add no value within the German context.

To explore whether this version of the questionnaire could be used in other European locations that also speak German and to account for German language variations in Switzerland and Austria, we conducted cognitive testing with one Swiss and one Austrian resident with master’s degrees in psychology who recommended that no changes needed to be made to the wording of items for use of the instrument in either country. This suggests that the questionnaire could be used with no modification in both Switzerland and Austria.

Compared to existing instruments measuring work-related well-being, the German TfWQ introduces a novel perspective within the landscape of German language work-related well-being measures, potentially enriching our understanding of how work itself can act as a resource. By focusing on thriving *through* work, the TfW adds a dimension that existing instruments like the COPSOQ and the Burnout Assessment Tool (BAT), which primarily focus on work demands and strains, often overlook. This approach aligns with a shift in organizational psychology towards more positive aspects of work life, such as mental, physical, and social functioning, which can promote a healthier and more productive workplace. Future empirical work is needed which uses the TfWQ in experimental/ field studies to evaluate its performance within applied research.

Our analyses confirmed that both the long and short forms of the questionnaire are reliable and valid. However, it’s important to note that some of the dimensions exhibited low empirical reliability, rendering them potentially unsuitable for use as standalone measures in their current form. Therefore, we recommend implementing the questionnaire in its entirety rather than selecting specific domains. Future research is necessary to improve the psychometric properties of the specific individual domains of TfW.

### Limitations and avenues for future research

Discrimination parameters of the long form showed moderate to high discrimination. The German TfW questionnaire is a 28-item instrument measuring six domains. It includes items that capture a wide variety of work-related well-being, some of which had only moderate discrimination (0.92–2.25). These items were retained because even though they don’t strongly differentiate among individuals, they are helpful to identify a range of attributes that are considered conceptually important as attributes for *TfW* [1]. In the original validation study conducted by Peters and colleagues [2], marginal discrimination parameters ranged from 0.66 to 2.92. Results are thus comparable between the German and English language versions.

The sample of this study consisted of 29.8% participants who worked in the police or armed forces, thus resulting in a slight sampling bias. However, the sample represented employees from 21 different sectors. Future studies could examine differences in *TfW* between different branches and differentiate between office-based and more operative jobs. Additionally, our sample size of *N* = 567 participants was slightly lower than the sample sizes recruited for similar studies. We recommend researchers using the German TfWQ in their research to report results of IRT models in their papers if they are using larger or more homogenous samples to further increase our confidence in the utility of the questionnaire. Since our sample was partially recruited from the researchers personal connections, mean age of the sample was relatively low (*M* = 31.46 years; *SD* = 9.17). We recommend future studies to be conducted on older employees and aim for an even more balanced gender ratio to improve generalizability. Furthermore, our sample was highly educated, future studies should specifically investigate the German TfWQ within blue-collar employees.

Due to parsimony, we selected single-item measures to capture trust in management, fatigue, and stress to assess convergent validity. Although the short scales we used stem from an established questionnaire, we recommend future studies to capture these validation constructs with longer measures to capture the full depth of these constructs.

## Conclusions

The current study showed that the German adaptation of the long and short forms of the TfW questionnaire are valid and reliable measures of work-related wellbeing. The questionnaire captures a broad spectrum of factors within work which contribute to employees overall thriving and well-being from and within their work which can also spill over to aspects of their private lives. This instrument provides a free resource for organizations to measure their employees’ work-related well-being with a well constructed and validated scale which can easily be implemented.

### Electronic supplementary material

Below is the link to the electronic supplementary material.

Supplementary Material 1

## Data Availability

The data can be obtained from the corresponding author upon reasonable request.
