# Supplementary material for: Thriving from work questionnaire: German translation and validation
Source: BMC Public Health. 2024 Jun 19;24:1634. doi: 10.1186/s12889-024-19037-0 (PMC11186060; doi:10.1186/s12889-024-19037-0)
Supplement: Supplementary file 1 — Supplementary Material 1 [file 12889_2024_19037_MOESM1_ESM.docx]

**TITLE PAGE**

**Thriving from Work Questionnaire: German Translation and Validation**

**Authors**

Stephanie M. Neidlinger* ^1,2^ (neidlinger@hsu-hh.de)

Susan E. Peters ^2^ (sepeters@hsph.harvard.edu)

Daniel A. Gundersen ^2,3, 4^ (daniel.gundersen@rutgers.edu)

Jörg Felfe ^1^ (felfe@hsu-hh.de)

**Affiliations:**

^1^ Department of Work, Organizational, and Business Psychology, Helmut-Schmidt University, Hamburg, 22043, Germany

^2^ Center for Work, Health, and Well-being, Harvard T.H. Chan School of Public Health, Boston, MA 02115, USA

^3^ Rutgers Institute for Nicotine and Tobacco Studies, New Brunswick, NJ, 08901, USA

^4^ Division of General Internal Medicine, Rutgers Robert Wood Johnson Medical School, New Brunswick NJ, 08901, USA

*Corresponding author and address

**Keywords**: Questionnaires, Worker Well-being, Item Response Theory, Study Validation, Germany

**APPENDIX:**

**APPENDIX A – Thriving from Work German Questionnaire**

**Table A1. German Thriving from Work questionnaire**.

| **Domain** | **#** | **Item** |  |
| --- | --- | --- | --- |
| Arbeitsbezogenes emotionales und psychologisches Wohlbefinden | **1** | **Ich liebe meinen Job.** |  |
|  | 2 | Meine Arbeit fügt meinem Leben einen Sinn hinzu. |  |
|  | 3 | Meine Arbeit ermöglicht es mir, mein volles Potenzial auszuschöpfen. |  |
|  | 4 | Die Art von Arbeit, die ich mache, macht mich glücklich. |  |
|  | 5 | Ich bin mit meiner Arbeit zufrieden. |  |
|  | 6 | Meine Arbeit trägt zu meiner allgemeinen Lebenszufriedenheit bei. |  |
| Soziales Wohlbefinden durch Arbeit | **7** | **Ich werde bei der Arbeit fair behandelt.** |  |
|  | 8 | Ich fühle mich von den Menschen, mit denen ich arbeite, unterstützt. |  |
|  | 9 | Ich fühle mich von den Menschen, mit denen ich zusammenarbeite, wertgeschätzt. |  |
|  | 10 | Ich werde bei der Arbeit mit Respekt behandelt. |  |
|  | 11 | Bei der Arbeit habe ich das Gefühl, dazuzugehören. |  |
| Integration von Arbeit und Privatleben | **12** | **Ich kann ein gesundes Gleichgewicht zwischen meiner Arbeit und meinem Leben außerhalb der Arbeit herstellen.** |  |
|  | 13 | Es gelingt mir leicht, meine Arbeit zu bewältigen und gleichzeitig auf meine Bedürfnisse und die Bedürfnisse meiner Familie einzugehen. |  |
|  | 14 | Ich fühle mich sicher auf dem Weg zur und von der Arbeit. |  |
| Grundbedürfnisse für Thriving | **15** | **Ich werde für die Arbeit, die ich mache, fair bezahlt.** |  |
|  | 16 | Ich bin zufrieden mit dem Umfang des bezahlten Urlaubs, den ich nehmen kann, um mich um mich oder Familienangehörige zu kümmern. |  |
|  | 17 | Ich habe das Gefühl, dass mein Arbeitsplatz sicher ist. |  |
|  | 18 | Ich habe gute Aufstiegschancen. |  |
| Arbeitsplatzgestaltung & Erfahrungen mit der Arbeit | **19** | **Ich bin zufrieden damit, wie viel Einfluss ich auf Entscheidungen habe, die meine Arbeit betreffen.** |  |
|  | 20 | Ich kann die Anforderungen meiner Arbeit leicht bewältigen. |  |
|  | 21 | Ich kann das Tempo meiner Arbeit gut kontrollieren. |  |
|  | 22 | Ich bin zufrieden damit, wie viel Kontrolle ich über meinen Tagesablauf habe. |  |
|  | 23 | Ich habe Zugang zu den Ressourcen, die ich brauche, um meine Arbeit gut zu machen. |  |
| Gesundheit und körperliches und psychisches Wohlergehen durch Arbeit | **24** | **Ich fühle mich bei der Arbeit psychologisch sicher.*** |  |
|  | **25** | **Ich fühle mich bei der Arbeit körperlich sicher.*** |  |
|  | 27 | Wenn ich die Arbeit verlasse, habe ich genug Energie, um die Dinge zu tun, die ich tun möchte oder muss. |  |
| Klima | **29** | **Ich kann bei der Arbeit Bedenken äußern oder Vorschläge machen, ohne Ärger zu bekommen.** |  |
|  | 30 | Ich erhalte bei der Arbeit Anerkennung für meine Leistungen. |  |

*Note*: *Select one of the two marked items for the short form questionnaire, both items are interchangeable. Peters and colleagues (2021) recommend to use the full scale and not individual domains. Items are to be scored on a six-point scale ranging from "Never" (0) to "Always" (5). Practitioners are advised to create a sum score for all items. For use in research, a model-based score is recommended. Items with bold numbers can be used as a short scale. Items 26 and 28 from domain “Gesundheit und körperliches und psychisches Wohlergehen durch Arbeit” were dropped from the German questionnaire due to low item intercorrelations and poor fit. Original item numbers were retained. Please see section “Scoring recommendations” in the discussion for information on scoring the questionnaire.

**APPENDIX B – Descriptive statistics and polychoric intercorrelations**

**Table B1. Descriptive Statistics for all Items.**

|  | **Item** | **Mean** | **SD** | **Min.** | **Max.** | **Skewness** | **Kurtosis** |  |  |
| --- | --- | --- | --- | --- | --- | --- | --- | --- | --- |
| **Work-related Emotional & Psychological Well-being** | | | | | | | | |  |
| 1 | I love my job. | 3.95 | 1.37 | 1 | 6 | -.420 | -.563 |  | |
| 2 | My work adds meaning to my life. | 3.81 | 1.40 | 1 | 6 | -.250 | -.812 |  |  |
| 3 | My job allows me to achieve my full potential. | 3.61 | 1.34 | 1 | 6 | -.116 | -.773 |  |  |
| 4 | The kind of work I do makes me happy. | 3.88 | 1.32 | 1 | 6 | -.242 | -.762 |  |  |
| 5 | I am satisfied with my job. | 4.13 | 1.22 | 1 | 6 | -.502 | -.350 |  |  |
| 6 | My work adds to my overall life satisfaction. | 3.84 | 1.30 | 1 | 6 | -.275 | -.663 |  |  |
| **Social Well-being from Work** | | | | | | | | | |
| 7 | I am treated fairly at work. | 4.49 | 1.17 | 1 | 6 | -.617 | -.059 |  |  |
| 8 | I feel supported by the people I work with. | 4.29 | 1.18 | 1 | 6 | -.453 | -.368 |  |  |
| 9 | I feel valued by the people I work with. | 4.32 | 1.24 | 1 | 6 | -.523 | -.333 |  |  |
| 10 | I am treated with respect at work. | 4.73 | 1.13 | 1 | 6 | -.869 | .447 |  |  |
| 11 | At work, I feel like I belong. | 4.39 | 1.25 | 1 | 6 | -.613 | -.267 |  |  |
| **Work-life Integration** | | | | | | | | | |
| 12 | I can achieve a healthy balance between my work and my life outside of work. | 3.99 | 1.36 | 1 | 6 | -.303 | -.747 |  |  |
| 13 | I can easily manage my job as well as attend to my needs and the needs of my family. | 3.86 | 1.27 | 1 | 6 | -.124 | -.682 |  |  |
| 14 | I feel safe getting to and from work. | 5.11 | 1.08 | 1 | 6 | -1.270 | 1.215 |  |  |
| **Basic Needs for Thriving** | | | | | | | | | |
| 15 | I am paid fairly for the job I do. | 4.31 | 1.53 | 1 | 6 | -.698 | -.480 |  |  |
| 16 | I am satisfied with the amount of paid leave I can take to care for myself or family members. | 4.20 | 1.48 | 1 | 6 | -.565 | -.613 |  |  |
| 17 | I feel my job is secure. | 4.96 | 1.21 | 1 | 6 | -1.255 | 1.139 |  |  |
| 18 | I have good opportunities for promotion. | 3.71 | 1.48 | 1 | 6 | -.215 | -.915 |  |  |
| **Job design & experience of work** | | | | | | | | | |
| 19 | I am happy with how much input I have in decisions that affect my work. | 3.83 | 1.30 | 1 | 6 | -.339 | -.678 |  |  |
| 20 | I can easily manage the demands of my job. | 4.27 | 1.05 | 1 | 6 | -.258 | -.225 |  |  |
| 21 | I have adequate control over the pace of my work. | 4.23 | 1.20 | 1 | 6 | -.527 | -.227 |  |  |
| 22 | I am happy with how much control I have over my work schedule. | 4.31 | 1.22 | 1 | 6 | -.565 | -.256 |  |  |
| 23 | I have access to the resources I need to do my job well. | 4.50 | 1.11 | 1 | 6 | -.623 | .131 |  |  |
| **Health, Physical, and Mental Well-being from Work** | | | | | | | | | |
| 24 | I feel psychologically safe at work. | 4.30 | 1.35 | 1 | 6 | -.538 | -.528 |  |  |
| 25 | I feel physically safe at work. | 5.05 | 1.16 | 1 | 6 | -1.290 | 1.365 |  |  |
| 26 | I feel excessive levels of stress from my work.** | 3.81 | 1.23 | 1 | 6 | -.413 | -.524 |  |  |
| 27 | After I leave work, I have enough energy to do the things I want or need to do. | 3.74 | 1.26 | 1 | 6 | -.169 | -.580 |  |  |
| 28 | I worry that I will get hurt at work.** | 5.12 | 1.20 | 1 | 6 | -1.422 | 1.219 |  |  |
| 29 | I can voice concerns or make suggestions at work without getting into trouble. | 4.40 | 1.27 | 1 | 6 | -.540 | -.381 |  |  |
| 30 | I receive recognition at work for my accomplishments. | 3.83 | 1.33 | 1 | 6 | -.185 | -.767 |  |  |

*Note*: *N* = 567. Items were ranked on a 6-point scale.

**Table B2. Polychoric correlations**

| **#** | **1** | **2** | **3** | **4** | **5** | **6** | **7** | **8** | **9** | **10** | **11** | **12** | **13** | **14** | **15** | **16** | **17** | **18** | **19** | **20** | **21** | **22** | **23** | **24** | **25** | **26** | **27** | **28** | **29** |
| --- | --- | --- | --- | --- | --- | --- | --- | --- | --- | --- | --- | --- | --- | --- | --- | --- | --- | --- | --- | --- | --- | --- | --- | --- | --- | --- | --- | --- | --- |
| **2** | .72 |  |  |  |  |  |  |  |  |  |  |  |  |  |  |  |  |  |  |  |  |  |  |  |  |  |  |  |  |
| **3** | .69 | .65 |  |  |  |  |  |  |  |  |  |  |  |  |  |  |  |  |  |  |  |  |  |  |  |  |  |  |  |
| **4** | .74 | .71 | .76 |  |  |  |  |  |  |  |  |  |  |  |  |  |  |  |  |  |  |  |  |  |  |  |  |  |  |
| **5** | .72 | .64 | .71 | .76 |  |  |  |  |  |  |  |  |  |  |  |  |  |  |  |  |  |  |  |  |  |  |  |  |  |
| **6** | .70 | .73 | .69 | .78 | .73 |  |  |  |  |  |  |  |  |  |  |  |  |  |  |  |  |  |  |  |  |  |  |  |  |
| **7** | .51 | .43 | .52 | .49 | .57 | .51 |  |  |  |  |  |  |  |  |  |  |  |  |  |  |  |  |  |  |  |  |  |  |  |
| **8** | .54 | .49 | .52 | .59 | .56 | .52 | .61 |  |  |  |  |  |  |  |  |  |  |  |  |  |  |  |  |  |  |  |  |  |  |
| **9** | .54 | .49 | .54 | .56 | .60 | .52 | .70 | .76 |  |  |  |  |  |  |  |  |  |  |  |  |  |  |  |  |  |  |  |  |  |
| **10** | .48 | .42 | .49 | .50 | .54 | .48 | .76 | .64 | .76 |  |  |  |  |  |  |  |  |  |  |  |  |  |  |  |  |  |  |  |  |
| **11** | .56 | .55 | .53 | .54 | .60 | .55 | .55 | .63 | .69 | .66 |  |  |  |  |  |  |  |  |  |  |  |  |  |  |  |  |  |  |  |
| **12** | .42 | .36 | .42 | .47 | .54 | .54 | .42 | .45 | .47 | .50 | .48 |  |  |  |  |  |  |  |  |  |  |  |  |  |  |  |  |  |  |
| **13** | .40 | .41 | .44 | .48 | .52 | .53 | .44 | .50 | .47 | .49 | .47 | .75 |  |  |  |  |  |  |  |  |  |  |  |  |  |  |  |  |  |
| **14** | .23 | .22 | .23 | .25 | .32 | .27 | .46 | .37 | .46 | .56 | .43 | .33 | .29 |  |  |  |  |  |  |  |  |  |  |  |  |  |  |  |  |
| **15** | .32 | .24 | .28 | .26 | .38 | .34 | .47 | .29 | .32 | .37 | .23 | .28 | .26 | .36 |  |  |  |  |  |  |  |  |  |  |  |  |  |  |  |
| **16** | .36 | .33 | .31 | .35 | .44 | .40 | .47 | .41 | .43 | .45 | .38 | .42 | .44 | .37 | .51 |  |  |  |  |  |  |  |  |  |  |  |  |  |  |
| **17** | .25 | .20 | .20 | .22 | .34 | .24 | .38 | .29 | .37 | .45 | .35 | .28 | .20 | .47 | .40 | .31 |  |  |  |  |  |  |  |  |  |  |  |  |  |
| **18** | .43 | .35 | .48 | .35 | .39 | .37 | .46 | .31 | .32 | .33 | .32 | .20 | .20 | .21 | .43 | .34 | .32 |  |  |  |  |  |  |  |  |  |  |  |  |
| **19** | .56 | .51 | .59 | .60 | .58 | .55 | .54 | .52 | .58 | .52 | .50 | .44 | .49 | .31 | .26 | .42 | .23 | .40 |  |  |  |  |  |  |  |  |  |  |  |
| **20** | .29 | .27 | .23 | .39 | .44 | .40 | .37 | .39 | .39 | .41 | .41 | .52 | .56 | .38 | .24 | .37 | .27 | .16 | .32 |  |  |  |  |  |  |  |  |  |  |
| **21** | .37 | .28 | .31 | .41 | .47 | .44 | .43 | .39 | .38 | .46 | .38 | .53 | .57 | .30 | .28 | .35 | .30 | .19 | .43 | .63 |  |  |  |  |  |  |  |  |  |
| **22** | .43 | .39 | .45 | .51 | .55 | .50 | .50 | .45 | .46 | .52 | .47 | .59 | .59 | .37 | .36 | .45 | .29 | .30 | .48 | .50 | .64 |  |  |  |  |  |  |  |  |
| **23** | .37 | .35 | .42 | .43 | .49 | .42 | .52 | .46 | .45 | .53 | .38 | .38 | .43 | .38 | .41 | .37 | .39 | .33 | .40 | .42 | .45 | .43 |  |  |  |  |  |  |  |
| **24** | .50 | .43 | .42 | .50 | .55 | .53 | .55 | .51 | .55 | .55 | .48 | .51 | .49 | .44 | .32 | .38 | .44 | .30 | .47 | .43 | .49 | .54 | .50 |  |  |  |  |  |  |
| **25** | .24 | .20 | .22 | .26 | .38 | .26 | .44 | .38 | .41 | .52 | .36 | .36 | .36 | .47 | .26 | .31 | .51 | .13 | .34 | .34 | .36 | .38 | .39 | .53 |  |  |  |  |  |
| **26** | .24 | .23 | .22 | .29 | .32 | .35 | .33 | .29 | .26 | .30 | .30 | .41 | .40 | .24 | .17 | .23 | .17 | .06 | .26 | .38 | .39 | .36 | .30 | .40 | .22 |  |  |  |  |
| **27** | .46 | .38 | .44 | .48 | .52 | .52 | .43 | .43 | .41 | .38 | .39 | .65 | .63 | .27 | .34 | .39 | .21 | .29 | .46 | .41 | .47 | .58 | .39 | .52 | .27 | .38 |  |  |  |
| **28** | -.11 | -.10 | -.08 | -.05 | .01 | .00 | .18 | .11 | .13 | .26 | .10 | .10 | .13 | .32 | .02 | .08 | .28 | -.19 | .03 | .08 | .15 | .12 | .19 | .22 | .54 | .31 | -.01 |  |  |
| **29** | .40 | .35 | .43 | .41 | .50 | .42 | .60 | .56 | .63 | .66 | .55 | .41 | .43 | .46 | .26 | .41 | .33 | .25 | .53 | .32 | .33 | .39 | .48 | .48 | .44 | .30 | .36 | .18 |  |
| **30** | .57 | .51 | .63 | .60 | .63 | .62 | .58 | .61 | .65 | .60 | .60 | .48 | .48 | .30 | .33 | .38 | .26 | .43 | .61 | .38 | .40 | .44 | .42 | .47 | .33 | .27 | .46 | .01 | .54 |
| Note: *N* = 567. Polychoric correlations displayed Since individual items are to be considered ordinal variables. Items 26 and 28 displayed the lowest intercorrelations and were removed from the German version of the Thriving from Work questionnaire. | | | | | | | | | | | | | | | | | | | | | | | | | | | | | |

**APPENDIX C ¬– Results of parametric bootstrapping simulations for all items of the short form.**

To investigate the model fit of the short form in more detail, we ran simulations using a parametric bootstrapping approach which simulate data under the model’s assumptions for all of the 8 items. If the simulated data closely resemble the observed data, it suggests that the model's assumptions are appropriate. The distribution of response categories for each item from the bootstrapped samples should closely match the statistics of the observed data if the model is an overall good fit. Specifically, we considered the model to be a good fit if the observed values were within the simulated confidence intervals.

We used the “itemfit” function from the “mirt” package to obtain item fit characteristics for all items in the short form to identify potential sources of model misfit. The chi square statistic and corresponding significant p-values indicate items 12 and 25 might cause the slightly higher RMSEA fit indices.

**Table C1. Item fit for the short form.**

|  | **Item** | ***χ2*** | **df** | **RMSEA** | ***p*** |
| --- | --- | --- | --- | --- | --- |
| 1 | I love my job. | 60.37 | 60 | 0.00 | 0.834 |
| 7 | I am treated fairly at work. | 43.64 | 54 | 0.00 | 0.842 |
| 12 | I can achieve a healthy balance between my work and my life outside of work. | 99.47 | 71 | 0.03 | 0.015 |
| 15 | I am paid fairly for the job I do. | 104.44 | 84 | 0.02 | 0.065 |
| 19 | I am happy with how much input I have in decisions that affect my work. | 75.84 | 61 | 0.02 | 0.096 |
| 20 | I can easily manage the demands of my job. | 72.23 | 56 | 0.02 | 0.071 |
| 24 | I feel psychologically safe at work. | 89.11 | 61 | 0.03 | 0.011 |
| 29 | I can voice concerns or make suggestions at work without getting into trouble. | 48.05 | 59 | 0.00 | 0.845 |
| *Note*. *N* = 567. | | | | | |

The results from parametric bootstrapping simulations showed no deviations from the simulated values to the observed values. All observed values were within the lower (2.5%) and higher (97.5%) bound of the simulated confidence intervals. In the context of assessing model fit, it's important to recognize that traditional cutoff values for model fit indices, such as RMSEA, have been criticized as arbitrary in the literature. While our model might yield slightly higher fit indices (e.g., RMSEA of 0.09), the results from our parametric bootstrapping simulation demonstrate the model's robustness and appropriateness. By closely aligning the simulated and observed values for all items, specifically in terms of the number of respondents in each response category, this study substantiates that our model is indeed a strong fit for the data. This validation not only strengthens our confidence in the model's statistical significance but also enhances the reliability of inferences, predictions, and decision-making based on the observed data, underscoring the utility of the model in our research.

**Table C2. Simulation results**

|  |  |  | **Response category** | | | | | |
| --- | --- | --- | --- | --- | --- | --- | --- | --- |
|  | **Item** | **CI boundary** | **1** | **2** | **3** | **4** | **5** | **6** |
| 1 | I love my job. | 2.5% | 23 | 40 | 97 | 116 | 139 | 53 |
|  |  | 97.5% | 46 | 66 | 135 | 155 | 181 | 83 |
|  |  | observed | 34 | 53 | 115 | 137 | 162 | 66 |
| 7 | I am treated fairly at work. | 2.5% | 2 | 23 | 53 | 131 | 166 | 101 |
|  |  | 97.5% | 11 | 45 | 84 | 173 | 211 | 138 |
|  |  | observed | 6 | 31 | 67 | 154 | 192 | 117 |
| 12 | I can achieve a healthy balance between my work and my life outside of work. | 2.5% | 14 | 53 | 91 | 111 | 135 | 68 |
|  |  | 97.5% | 32 | 83 | 127 | 150 | 175 | 100 |
|  |  | observed | 22 | 68 | 113 | 134 | 150 | 80 |
| 15 | I am paid fairly for the job I do. | 2.5% | 32 | 30 | 56 | 98 | 122 | 130 |
|  |  | 97.5% | 57 | 54 | 88 | 134 | 162 | 174 |
|  |  | observed | 43 | 40 | 70 | 115 | 145 | 154 |
| 19 | I am happy with how much input I have in decisions that affect my work. | 2.5% | 16 | 66 | 80 | 138 | 141 | 31 |
|  |  | 97.5% | 34 | 96 | 114 | 182 | 182 | 57 |
|  |  | observed | 24 | 83 | 100 | 161 | 156 | 43 |
| 20 | I can easily manage the demands of my job. | 2.5% | 1 | 11 | 96 | 166 | 153 | 52 |
|  |  | 97.5% | 9 | 29 | 133 | 210 | 196 | 82 |
|  |  | observed | 4 | 18 | 109 | 192 | 178 | 66 |
| 24 | I feel psychologically safe at work. | 2.5% | 4 | 7 | 26 | 82 | 121 | 244 |
|  |  | 97.5% | 15 | 21 | 48 | 117 | 162 | 292 |
|  |  | observed | 9 | 13 | 35 | 97 | 144 | 269 |
| 29 | I can voice concerns or make suggestions at work without getting into trouble. | 2.5% | 5 | 23 | 73 | 116 | 149 | 109 |
|  |  | 97.5% | 17 | 47 | 106 | 154 | 190 | 148 |
|  |  | observed | 11 | 35 | 91 | 135 | 169 | 126 |
| *Note*. *N* = 567. | | | | | | | | |

**APPENDIX D ¬– Transformation of Discrimination Parameters into Factor Loadings**

**Table D1: Factor loadings long form.**

| # | Item | General Thriving from Work | 1 | 2 | 3 | 4 | 5 | 6 |  |
| --- | --- | --- | --- | --- | --- | --- | --- | --- | --- |
| **Work-related Emotional & Psychological Well-being** | | | | | | | | | |
| 1 | I love my job. | 0.67 | 0.55 |  |  |  |  |  |  |
| 2 | My work adds meaning to my life. | 0.61 | 0.58 |  |  |  |  |  |  |
| 3 | My job allows me to achieve my full potential. | 0.66 | 0.52 |  |  |  |  |  |  |
| 4 | The kind of work I do makes me happy. | 0.72 | 0.56 |  |  |  |  |  |  |
| 5 | I am satisfied with my job. | 0.78 | 0.41 |  |  |  |  |  |  |
| 6 | My work adds to my overall life satisfaction. | 0.73 | 0.49 |  |  |  |  |  |  |
| **Social Well-being from Work** | | | | | | | | | |
| 7 | I am treated fairly at work. | 0.77 |  | 0.27 |  |  |  |  |  |
| 8 | I feel supported by the people I work with. | 0.75 |  | 0.38 |  |  |  |  |  |
| 9 | I feel valued by the people I work with. | 0.77 |  | 0.54 |  |  |  |  |  |
| 10 | I am treated with respect at work. | 0.80 |  | 0.34 |  |  |  |  |  |
| 11 | At work, I feel like I belong. | 0.73 |  | 0.29 |  |  |  |  |  |
| **Work-life Integration** | | | | | | | | | |
| 12 | I can achieve a healthy balance between my work and my life outside of work. | 0.71 |  |  | 0.52 |  |  |  |  |
| 13 | I can easily manage my job as well as attend to my needs and the needs of my family. | 0.72 |  |  | 0.52 |  |  |  |  |
| 14 | I feel safe getting to and from work. | 0.56 |  |  | – 0.16 |  |  |  |  |
| **Basic Needs for Thriving** | | | | | | | | | |
| 15 | I am paid fairly for the job I do. | 0.47 |  |  |  | 0.81 |  |  |  |
| 16 | I am satisfied with the amount of paid leave I can take to care for myself or family members. | 0.61 |  |  |  | 0.30 |  |  |  |
| 17 | I feel my job is secure. | 0.49 |  |  |  | 0.26 |  |  |  |
| 18 | I have good opportunities for promotion. | 0.49 |  |  |  | 0.29 |  |  |  |
| **Job design & experience of work** | | | | | | | | | |
| 19 | I am happy with how much input I have in decisions that affect my work. | 0.73 |  |  |  |  | – 0.05 |  |  |
| 20 | I can easily manage the demands of my job. | 0.60 |  |  |  |  | 0.44 |  |  |
| 21 | I have adequate control over the pace of my work. | 0.63 |  |  |  |  | 0.67 |  |  |
| 22 | I am happy with how much control I have over my work schedule. | 0.72 |  |  |  |  | 0.31 |  |  |
| 23 | I have access to the resources I need to do my job well. | 0.66 |  |  |  |  | 0.13 |  |  |
| **Health, Physical, and Mental Well-being from Work** | | | | | | | | | |
| 24 | I feel psychologically safe at work. | 0.75 |  |  |  |  |  | 0.16 |  |
| 25 | I feel physically safe at work. | 0.57 |  |  |  |  |  | 0.68 |  |
| 27 | After I leave work, I have enough energy to do the things I want or need to do. | 0.67 |  |  |  |  |  | – 0.14 |  |
| 29 | I can voice concerns or make suggestions at work without getting into trouble. | 0.72 |  |  |  |  |  |  |  |
| 30 | I receive recognition at work for my accomplishments. | 0.78 |  |  |  |  |  |  |  |

*Note*: *N* = 567.

**Table D2. Factor loadings short form.**

| Long form item # | Item | Loadings |
| --- | --- | --- |
| 1 | I love my job. | 0.65 |
| 7 | I am treated fairly at work. | 0.81 |
| 12 | I can achieve a healthy balance between my work and life outside of work. | 0.65 |
| 15 | I am paid fairly for the job I do. | 0.48 |
| 19 | I am happy with how much input I have in decisions that affect my work. | 0.73 |
| 20 | I can easily manage the demands of my job. | 0.56 |
| 24 | I feel psychologically safe at work. | 0.56 |
| 29 | I can voice my concerns or make suggests at work without getting into trouble. | 0.73 |
| *Note: N* = 567. | | |

Tables D1 and D2 showcase the loadings of each item on the general factor and specific factors, derived from the mirt “summary()” function. These loadings are analogous to factor loadings in structural equation modeling but are obtained from an IRT-based bifactor analysis of our models. The values can be interpreted similarly to CFA factor loadings. It must be noted that since these values were obtained from the IRT model, factor loadings from a full CFA performed on its own, may vary. The purpose of this table is to facilitate interpretation for researchers unaccustomed with IRT.

**APPENDIX E ¬– Scoring recommendations and correlations between sum scores for Thriving from Work with the validation constructs.**

**Table E1. Correlations between sum scores of Thriving from Work and the validation constructs.**

|  | *TfW* long from | *TfW* short from |
| --- | --- | --- |
| *TfW* short from | .96*** |  |
| Life Satisfaction | .67*** | .66*** |
| Trust in Management | .59*** | .59*** |
| Well-being | .65*** | .62*** |
| Fatigue | -.42*** | -.41*** |
| Stress | -.38*** | -.38*** |
| Phone calls | .09^*^ | .08^†^ |
| Note: *N* = 567.^†^ p < .10, * *p* < .05. **** *p* < .01. ***** *p* < .001. | | |

*Scoring Recommendations*

We recommend scoring both the long and short form of the German Thriving from Work questionnaire with a model-based score which can be obtained from the bifactor model in item response theory (IRT) and can provide a more nuanced and accurate assessment of latent traits. Model-based scoring is recommended for the questionnaire’s use in research. To ensure practicability for use of practitioners who are not trained in IRT and complex statistical software, we also computed sum scores for both the long and short form of the German Thriving from Work questionnaire. Correlations between both scoring methods with the validation constructs were comparable and showed no noteworthy differences. To obtain sum scores, response valued for all items in the questionnaires are summed, thus retaining the original information from each item in the questionnaire.
